# Individual radiosensitivity in a breast cancer collective is changed with the patients’ age

**DOI:** 10.2478/raon-2013-0061

**Published:** 2014-01-22

**Authors:** Judith Auer, Ulrike Keller, Manfred Schmidt, Oliver Ott, Rainer Fietkau, Luitpold V. Distel

**Affiliations:** Department of Radiation Oncology of the University Hospitals and Friedrich-Alexander-University of Erlangen-Nürnberg, Erlangen, Germany

**Keywords:** individual radiosensitivity, chromosomal aberrations, age, fluorescence *in situ* hybridization, radiotherapy, breast cancer

## Abstract

**Background:**

Individual radiosensitivity has a crucial impact on radiotherapy related side effects. Our aim was to study a breast cancer collective for its variation of individual radiosensitivity depending on the patients’ age.

**Materials and methods:**

Peripheral blood samples were obtained from 129 individuals. Individual radiosensitivity in 67 breast cancer patients and 62 healthy individuals was estimated by 3-color fluorescence *in situ* hybridization.

**Results:**

Breast cancer patients were distinctly more radiosensitive compared to healthy controls. A subgroup of 9 rather radiosensitive and 9 rather radio-resistant patients was identified. A subgroup of patients aged between 40 and 50 was distinctly more radiosensitive than younger or older patients.

**Conclusions:**

In the breast cancer collective a distinct resistant and sensitive subgroup is identified, which could be subject for treatment adjustment. Preliminary results indicate that especially in the range of age 40 to 50 patients with an increased radiosensitivity are more frequent and may have an increased risk to suffer from therapy related side effects.

## Introduction

The very demanding task of a radiotherapy is to kill all cancer cells and at the same time to spare the surrounding normal tissue. However, there is a spectrum of confounding parameters which can affect normal tissue tolerance. One parameter is the individual radiosensitivity of the patient.^1^ By prediction of individual radiosensitivity and adjusting the dosing regimen side effects and adverse events could be avoided. Different techniques to determine individual radiosensitivity were used with varying degree of success.^2^–^5^ Yet, chromosomal aberrations are generally known to have the potential to predict individual radiosensitivity.^1^,^6^–^10^ Mostly lymphocytes are irradiated *in vitro* in the G0 phase and afterwards stimulated to proceed in cell cycle. The advantage of this approach is that chromosomal aberrations are a late endpoint in radiation damage processing. Chromosomal aberrations cover the entire ability of cells to recover from DNA damage and to process these damages. It includes: (i) DNA damage repair, (ii) mediating a proper signal transduction, (iii) achieving an appropriate cell cycle control and (iiii) induce cell death, if necessary. The analysis of this late endpoint could be favorable compared to assays detecting earlier endpoints like DNA-double strand breaks (γH2AX), apoptosis (sub-G1 peak, Annexin V or cleaved Caspase 3) or cell-cycle control (PI-flow cytometry).

Nevertheless, even if the individual radiosensitivity could be determined with great accuracy, it is not certain that a study allows proving the relation between radiosensitivity and side effect. There have been several studies with disappointing results. The reason is based on the observations of Jung *et al*.^11^ that the probability to develop adverse events is defined depending on radiotherapy treatment. The treatment defined risk will be higher in the group of sensitive individuals than in the intermediate and resistant group. Nevertheless, also individuals in the intermediate group take the risk of developing side effects and because this group is normally much bigger than the sensitive one there will be more individuals suffering from side effects in the intermediate group than in the sensitive group. As a consequence, testing predictive assays individuals must be grouped prospectively in sensitive, intermediate and radio-resistant and side effects may be collected at different time points.^2^

Our aim was to determine the sensitive, intermediate and resistant groups of a breast cancer collective by a 3-color-fluorescence *in situ* hybridization approach (3-color-FISH) in a prospective study. Therefore the frequency of chromosomal aberrations in blood lymphocytes after *in vitro* irradiation with 2 Gy was detected. Additionally the influence of age on individual radiosensitivity was studied.

## Material and methods

67 breast cancer patients and 62 healthy controls were included in the prospective study (Table 1). Patients were included into the study randomly. A peripheral blood sample was obtained from patients and healthy controls. The blood withdrawal was performed within one week before the beginning of radiotherapy. Blood samples were divided into two parts. One part was used to detect spontaneous aberrations, the other one was irradiated *in vitro* with 2 Gy on a 6 MV linear accelerator (Mevatron, Siemens, Germany). This study was approved by the ethics review committees of the Friedrich-Alexander-Universität Erlangen-Nürnberg (No. 2725), and informed consent was obtained from all patients and healthy volunteers. None of the patients used medications which are suspected to increase radiosensitivity.

Chromosomal aberrations were detected using 3-color FISH with whole chromosome painting of chromosomes 1, 2 and 4 as described earlier.^7^,^12^ Heparinized blood samples of each patient were cultivated at 37°C for 48 h in RPMI 1640 medium with 1% penicillin-streptomycin, 1% glutamine, 2.5% phytohemagglutinin and 15% fetal calf serum. Cell division was stopped by adding colcemid (5μg/ml) 2.5 h before starting preparation of lymphocytes according to a standard technique as previously described.^13^ Metaphase spreads were dropped on slides and preserved in 70% ethanol at −20°C before performing the 3-color FISH assay with whole chromosome painting of chromosomes 1, 2 and 4.^12^ Chromosomes were counterstained with DAPI.^14^

**FIGURE 1:.**
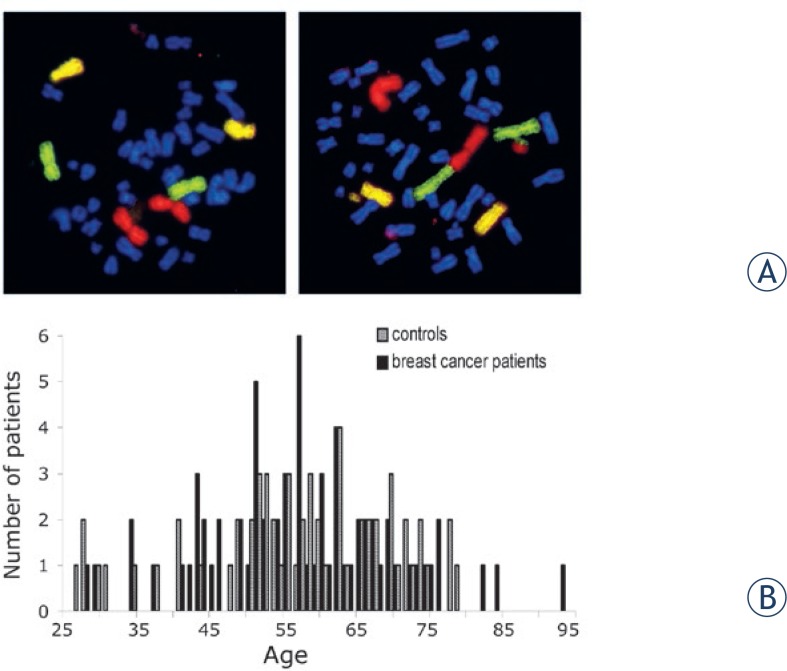
**A** – Three-color FISH painting of chromosomes 1 (red), 2 (green) and 4 (yellow). A metaphase without aberrations (left) and a metaphase (right) with a reciprocal dicentric chromosome and a break are displayed. The two aberrations were scored as 3 breaks. **B** – Frequency of patients and controls age distribution.

A fluorescence microscope (Zeiss, Germany) was used to detect chromosomal aberrations, such as breaks, deletions, translocations, dicentrics, insertions and rings (Figure 1 A). 500 metaphases were scored for non-irradiated controls and 150 metaphases for 2 Gy *in vitro* irradiated slides.^15^ Additionally the mitotic index was counted by using image analysis software (Biomas 3.3, MSAB, Erlangen, Germany).

Data analysis and statistics were performed using SPSS 16 for Windows (IBM Corp., Armonk, NY, USA). For comparing the results referred to the different groups, the Kolmogorov–Smirnov test and Lilliefors test were applied for testing normality and data were fitted by a Gaussian distribution. Standard deviations of the Gaussian distributions have been used to designate an individual’s categorization. The cut-off values of 2 and 3 standard deviations are equivalent to the 95% and 99% confidence intervals. Different groups were compared using the two-sample t-test. Graphics were plotted with TechPlot for Windows 3.0.11 (SFTek, Dr. Ralf Dittrich, Braunschweig, Germany).

## Results

Our intention was to study the individual radiosensitivity in a prospective collective of breast cancer patients. Three-color-FISH was used to estimate radiosensitivity (Figure 1 A). All aberrations in chromosomes 1, 2 and 4 were scored for themselves and additionally the breaks per metaphase were estimated according to the theoretically necessary number of breaks to form the aberrations: fragments equate to one break event, reciprocal translocations, simple dicentrics and rings equate to two break events and complex aberrations to as many breaks would be needed to cause this aberration at least. The 129 individuals in this study consisted of 67 patients suffering from breast cancer and 62 healthy individuals as controls. The median age of the breast cancer patients was 57.2a (±12.9a) and 56.3a (±14.2a) for the control individuals (Figure 1 B). The patients’ clinical characteristics are described in Table 1. All patients got adjuvant radiotherapy and were irradiated with single doses of 1.8 Gy up to a total dose of 50.4 Gy. Blood was analyzed after *in vitro* irradiation with 2 Gy.

**FIGURE 2.**
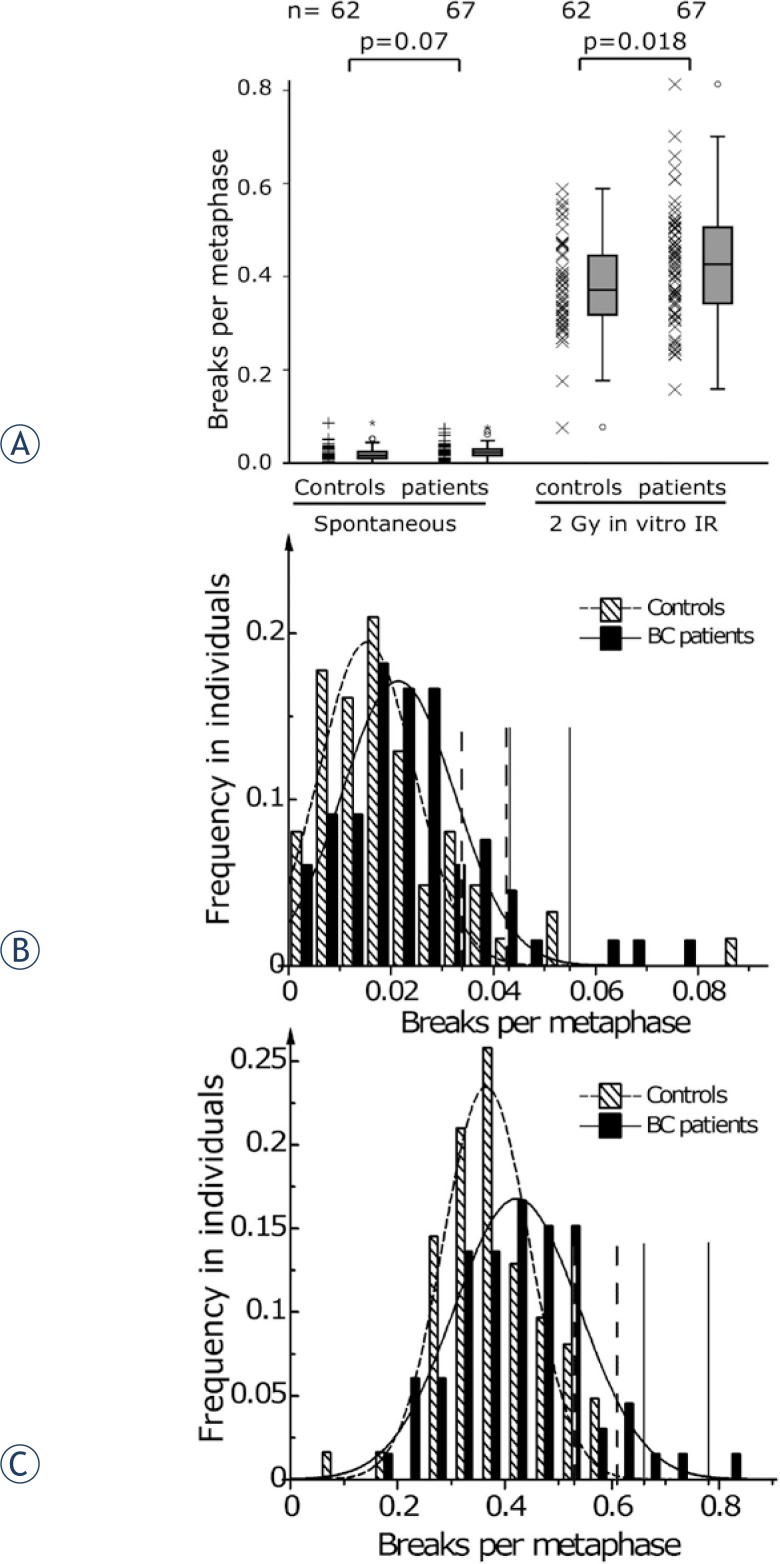
Individual chromosomal radiosensitivity as measured in *in vitro* and *in vivo* irradiated lymphocytes by three-color FISH. Chromosomal aberrations were scored as breaks per metaphase. Blood samples were derived from healthy individuals (controls) and breast cancer patients (BC patients). **A** – Breaks per metaphase of unirradiated samples (spontaneous) and after *in vitro* irradiation by a dose of 2 Gy (2 Gy *in vitro* IR) were shown as scatter plots and box plots. Frequency of breaks per metaphase fitted to a Gaussian distributions of **B** – unirradiated samples, **C** - after *in vitro* irradiation by a dose of 2 Gy

If possible, 650 metaphases per patient were scored, *i.e*. 150 metaphases from lymphocytes irradiated *in vitro* with 2 Gy and 500 metaphases from non-irradiated lymphocytes. Breaks per metaphase in the patients’ group were slightly higher than in the group of healthy individuals. An *in vitro* irradiation with a 2 Gy dose leads to a distinct higher amount of breaks. Yet there was only a trend to an increase of breaks in the patients’ group compared to the controls (Figure 2 A).

Breaks per metaphases were classified and the normality of the distributions was tested by the Kolmogorov–Smirnov algorithm. All distributions were found to be normally distributed and Gaussian fits were performed. The average of the normal distribution of unirradiated blood samples was displaced to higher values by 23% in the breast cancer group compared to the healthy individuals group (Figure 2 B). The distributions of the blood samples after 2 Gy *in vitro* irradiation were changed similarly. The mean of the distribution was shifted by 12% to higher breaks per metaphase and the width of the distribution increased by 28% (Figure 2 C).

**FIGURE 3.**
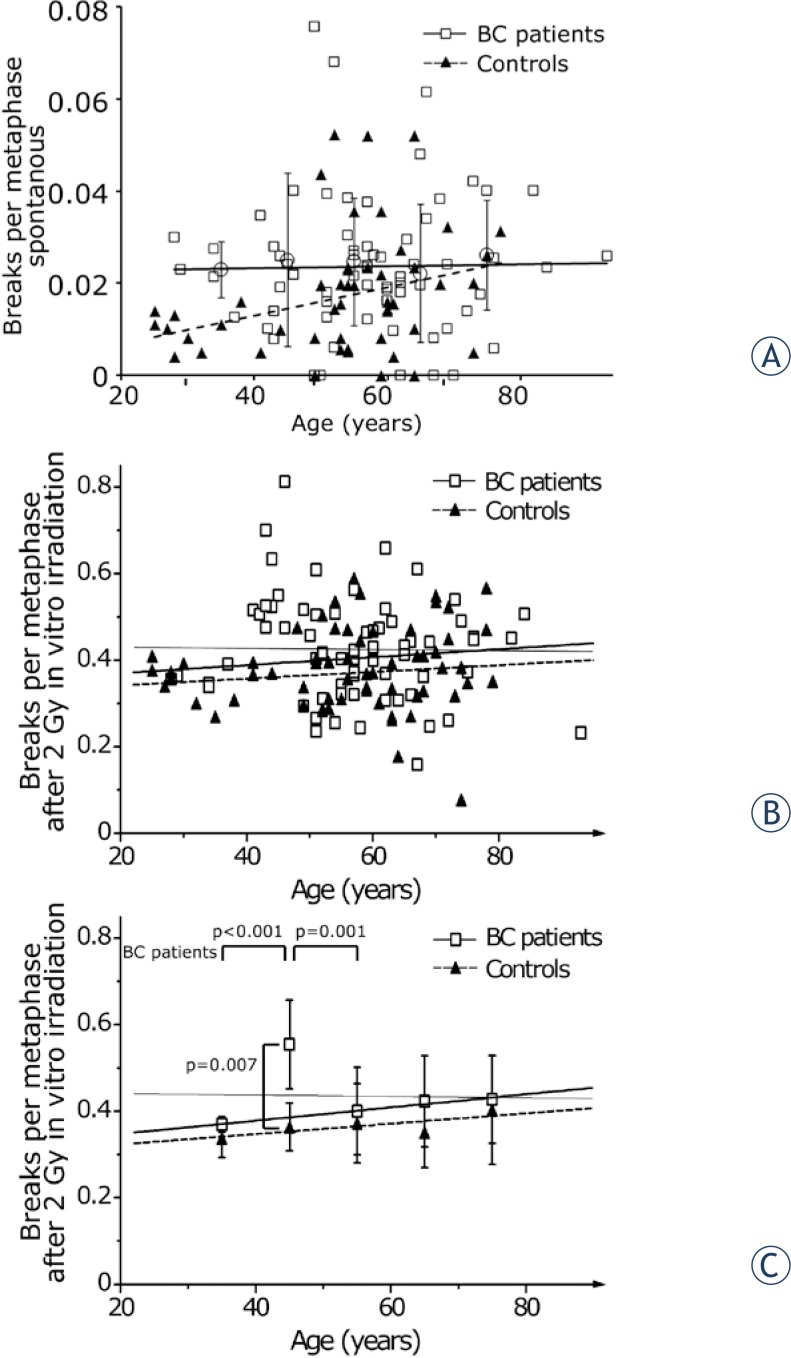
Breaks per metaphase in dependence of the patients (open square, continuous line) and healthy individuals age (filled triangle, dashed line). Ten year intervals were summarized and the average was given as mean value with its standard deviation. **A** – Unirradiated blood samples, **B** – samples after 2 Gy *in vitro* irradiation individual values and **C** – samples after 2 Gy *in vitro* irradiation mean values and standard deviation. B C – Thin line gives the linear regression including all patients values, thick continuous line gives the linear regression excluding the group aged 40 to 50 years.

Additionally we studied the age dependence on the breaks per metaphase. A broad range of patients between 28 and 93 years was analyzed. All values of ten year intervals were summarized and the average was given as mean value. Breaks per metaphase did not increase with patients’ age in the unirradiated group. In healthy controls breaks per metaphase increased with age (Figure 3 A). After *in vitro* irradiation by 2 Gy a linear regression indicated a marginal decrease of break events with advancing age of the patients and a distinct increase of the healthy controls breaks per meta-phases (Figure 3 B). However, there was a distinct deviation of the 40 to 50 years data point from the linear regression and marked it as an outlier (Figure 3 B, C). Patients aged between 40 and 50 years have a significantly increased level of breaks per metaphase. After exclusion of the patients in this age range the linear regression matches the remaining data points and indicates an increase of breaks with advancing age by 0.01 breaks per metaphase per ten years.

**FIGURE 4.**
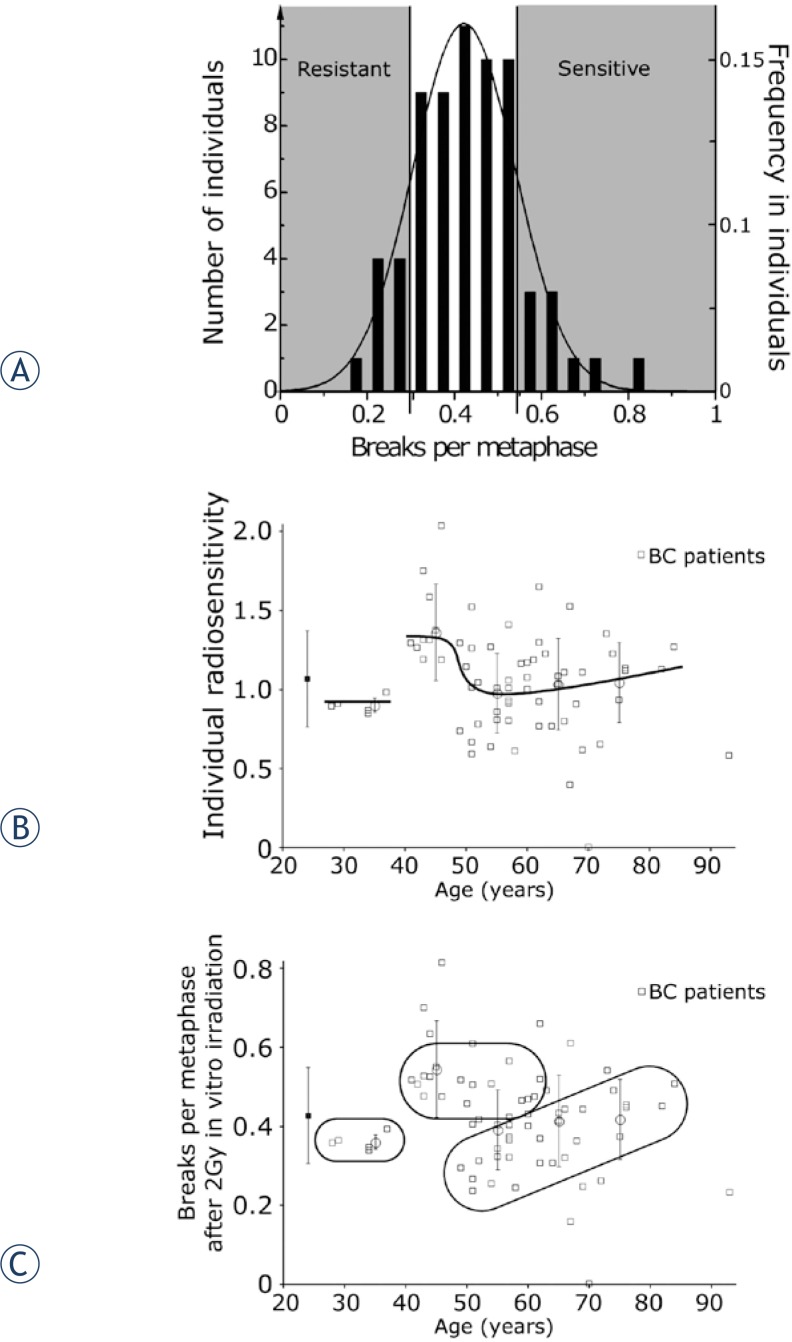
**A** - Classification of the breast cancer patients on the basis of the breaks per metaphase level after *in vivo* irradiation (2 Gy IR). **B** – Putative individual radiosensitivity dependent on breast cancers patients’ age. **C** - Presumed classification of the breast cancer patients in three groups of different radiosensitivity.

Breaks per metaphases were fitted in a Gaussian distribution and patients were classified in resistant, intermediate and sensitive patients (Figure 4 A). In this way we defined a group of 49 intermediate patients, 9 resistant and 9 sensitive patients. A putative age dependency is displayed in Figure 4 B. Patients were divided into three groups which were supposed to reflect the patients’ sensitivities best (Figure 4 C).

## Discussion

Chromosomal aberrations were detected in peripheral blood lymphocytes of 67 breast cancer patients and 62 healthy controls. Blood samples were taken before starting adjuvant radiotherapy using a 2 Gy *in vitro* irradiation. Breaks per metaphase were normally distributed with increased values of the breast cancer patients compared to controls. It reflects that individuals with an increased probability of suffering from chromosomal aberrations or other mutations have an increased risk of cancer.^16^,^17^ However, the main interest was not the comparison between the radiosensitivity of control individuals and cancer patients, but the grouping into radioresistant to sensitive cancer patients. These patient groups should be surveyed for the next years and it should be estimated whether in the sensitive group a higher portion of patients suffering from chronic side effects will be found^18^,^19^.

The dose of 2 Gy given in the *in vitro* irradiation leads to a distinctly increased rate of breaks per metaphase. Breaks per metaphases fitted very well in a Gaussian distribution and were classified in resistant, intermediate and sensitive patients (Figure 4 A). The patients will be surveyed over the next years to gather the occurrence of late therapy side effects. However, the risk for developing radiation-induced late effects after conventional RT for breast cancer is low. Fibrosis and telangiectasia are still the most common late effects. Due to the low frequency and the time delay of occurrence of the adverse events it needs several years to quantify these late effects properly.^20^ Therefore, we cannot compare the individual radiosensitivity in means of breaks per metaphase with late effects so far.

A further aspect of this study was the age dependency of radiosensitivity. There is a very limited increase of spontaneous chromosomal aberrations with advanced age. It is in contrast to a study comparing young healthy individuals (38.8 years) with older individuals (69.2 years) and a spontaneous increase of chromosomal aberrations by a factor of 5.^21^ After a 2 Gy *in vitro* irradiation there is a discontinuous course of metaphase breaks per patient in dependence of age. Patients with age 40 to 50 are distinctly more sensitive. A putative age dependent radiosensitivity is displayed in Figure 4 B. Very young patients below age 40 have a low average radiosensitivity, patients between 40 and 50 years are extraordinarily sensitive and patients older than 50 are distinctly less sensitive. With further increasing age the sensitivity slowly increases (Figure 4 B). The reason for this may be that the cancer at different ages is acquired for different reasons. The very young patients have acquired the cancer by other mechanisms than the other groups, probably by an immature tissue. Patients between 40 and 50 are a group of sensitive individuals and may have acquired cancer because of genetic factors influencing the early onset of breast cancer. About 5–7 percent of all breast cancer patients have a familial breast cancer history. The most common mutations linked to an earlier onset of cancer are the BRCA 1 and 2 mutations and a large number of additional genes including STK11, CDH1, PTEN, TP53.^22^ Genes related to an early onset of cancer are frequently related to an increased sensitivity to ionizing radiation and this may be the reason for the observed higher number of chromosomal breaks. Individuals with a resistant or intermediate sensitivity do not acquire cancer before the age of 50 due to the lower mutation probability.

In Figure 4 C the three putative groups are marked. It must be assumed that patients in the sensitive group have an increased probability to suffer from therapy related side effects. However, the risk for radiation therapy related side effects in breast cancer are generally low, therefore only a limited increase in additional side effects will be expected. There are some publications that mention an increased risk in younger patients after radiotherapy and chemotherapy treatment.^23^–^25^ It’s worth bearing in mind that in this age group sensitive patients exists and therapy related side effects should be monitored carefully.

## Conclusions

In the breast cancer collective a distinctly resistant and sensitive subgroup is identified by using 3-color-FISH after 2 Gy *in vitro* irradiation of peripheral blood lymphocytes, which could be subject for treatment adjustment. Especially in the range of age 40 to 50 there is an increased fraction of patients having an increased radiosensitivity. These patients have an increased probability to suffer from therapy related side effects.

## Figures and Tables

**TABLE 1.** Patients characteristics

	**129 individuals**	
	**67 breast cancer patients**	**62 healthy controls**
mean age	57.2a (±12.9a)	56.3a (±14.2a)
stage 0 / I / IIA / IIB / IV	5 / 30 / 11 / 18 / 3	
Tis / T1 / T2 / T3 / T4	6 / 37 / 22 / 1 / 1	-
N0 / N+	44 / 23	-
M0 / M1	64 / 3	-
Dcis / no Dcis	37 / 30	-
mastectomy	9	-
breast-preserving	58	
single dose / total dose	1.8 Gy / 50.4 Gy	
body weight	78.8 kg (±15.4 kg)	
volume 20%–95% isodose	1460 ml (±870 ml)	-
dose*volume	6.8 Gy*dm^3^ (±4.5 Gy dm^3^)	

## References

[1] Huber R, Braselmann H, Geinitz H, Jaehnert J, Baumgartner A, Thamm R (2011). Chromosomal radiosensitivity and acute radiation side effects after radiotherapy in tumour patients--a follow-up study. Radiat Oncol.

[2] Dikomey E, Borgmann K, Peacock J, Jung H (2003). Why recent studies relating normal tissue response to individual radiosensitivity might have failed and how new studies should be performed. Int J Radiat Oncol Biol Phys.

[3] Zschenker O, Raabe A, Boeckelmann IK, Borstelmann S, Szymczak S, Wellek S (2010). Association of single nucleotide polymorphisms in ATM, GSTP1, SOD2, TGFB1, XPD and XRCC1 with clinical and cellular radiosensitivity. Radiother Oncol.

[4] Lopez E, Guerrero R, Nunez MI, del Moral R, Villalobos M, Martinez-Galan J (2005). Early and late skin reactions to radiotherapy for breast cancer and their correlation with radiation-induced DNA damage in lymphocytes. Breast Cancer Res.

[5] Ambrosone CB, Tian C, Ahn J, Kropp S, Helmbold I, von Fournier D (2006). Genetic predictors of acute toxicities related to radiation therapy following lumpectomy for breast cancer: a case-series study. Breast Cancer Res.

[6] Borgmann K, Haeberle D, Doerk T, Busjahn A, Stephan G, Dikomey E (2007). Genetic determination of chromosomal radiosensitivities in G0- and G2-phase human lymphocytes. Radiother Oncol.

[7] Distel LV, Neubauer S, Keller U, Sprung CN, Sauer R, Grabenbauer GG (2006). Individual differences in chromosomal aberrations after in vitro irradiation of cells from healthy individuals, cancer and cancer susceptibility syndrome patients. Radiother Oncol.

[8] Pantelias GE, Terzoudi GI (2011). A standardized G2-assay for the prediction of individual radiosensitivity. Radiother Oncol.

[9] Scott D (2000). Chromosomal radiosensitivity, cancer predisposition and response to radiotherapy. Strahlenther Onkol.

[10] Scott D (2004). Chromosomal radiosensitivity and low penetrance predisposition to cancer. Cytogenet Genome Res.

[11] Jung H, Beck-Bornholdt HP, Svoboda V, Alberti W, Herrmann T (2001). Quantification of late complications after radiation therapy. Radiother Oncol.

[12] Neubauer S, Dunst J, Gebhart E (1997). The impact of complex chromosomal rearrangements on the detection of radiosensitivity in cancer patients. Radiother Oncol.

[13] Neubauer S, Gebhart E, Schmitt G, Birkenhake S, Dunst J (1996). Is chromosome in situ suppression (CISS) hybridization suited as a predictive test for intrinsic radiosensitivity in cancer patients?. Int J Oncol.

[14] Keller U, Grabenbauer G, Kuechler A, Sprung C, Müller E, Sauer R (2005). Cytogenetic instability in young patients with multiple primary cancers. Cancer Genet Cytogenet.

[15] Keller U, Grabenbauer G, Kuechler A, Sauer R, Distel L (2004). Radiation sensitivity testing by FISH: how many metaphases have to be analysed?. Int J Radiat Biol.

[16] Baeyens A, Van Den Broecke R, Makar A, Thierens H, De Ridder L, Vral A (2005). Chromosomal radiosensitivity in breast cancer patients: influence of age of onset of the disease. Oncol Rep.

[17] Vineis P (2004). Individual susceptibility to carcinogens. Oncogene.

[18] Oblak I, Petric P, Anderluh F, Velenik V, Fras PA (2012). Long term outcome after combined modality treatment for anal cancer. Radiol Oncol.

[19] Trost N, Juvan P, Sersa G, Debeljak N (2012). Contrasting effect of recombinant human erythropoietin on breast cancer cell response to cisplatin induced cytotoxicity. Radiol Oncol.

[20] Ott OJ, Hildebrandt G, Potter R, Hammer J, Lotter M, Resch A (2007). Accelerated partial breast irradiation with multi-catheter brachytherapy: Local control, side effects and cosmetic outcome for 274 patients. Results of the German-Austrian multi-centre trial. Radiother Oncol.

[21] Mladinic M, Kopjar N, Milic M, Dasovic AB, Huzak M, Zeljezic D (2010). Genomic instability in a healthy elderly population: a pilot study of possible cytogenetic markers related to ageing. Mutagenesis.

[22] Melchor L, Benitez J (2013). The complex genetic landscape of familial breast cancer. Hum Genet.

[23] Braithwaite D, Moore DH, Satariano WA, Kwan ML, Hiatt RA, Kroenke C (2012). Prognostic impact of comorbidity among long-term breast cancer survivors: results from the LACE study. Cancer Epidemiol Biomarkers Prev.

[24] Demoor-Goldschmidt C, Supiot S, Mahe MA (2012). Breast cancer after radiotherapy: Risk factors and suggestion for breast delineation as an organ at risk in the prepuberal girl. Cancer Radiother.

[25] Huang YS, Lee CC, Chang TS, Ho HC, Su YC, Hung SK (2011). Increased risk of stroke in young head and neck cancer patients treated with radiotherapy or chemotherapy. Oral Oncol.

